# Inequalities in global health inequalities research: A 50-year bibliometric analysis (1966-2015)

**DOI:** 10.1371/journal.pone.0191901

**Published:** 2018-01-31

**Authors:** Lucinda Cash-Gibson, Diego F. Rojas-Gualdrón, Juan M. Pericàs, Joan Benach

**Affiliations:** 1 Health Inequalities Research Group, Employment Conditions Knowledge Network (GREDS-EMCONET), Department of Political and Social Sciences, Universitat Pompeu Fabra, Barcelona (Catalonia, Spain); 2 Johns Hopkins University—Pompeu Fabra University Public Policy Center, Barcelona (Catalonia, Spain); 3 Faculty of Medicine, CES University, Medellín (Antioquia, Colombia); 4 School of Graduate Studies, CES University, Medellín (Antioquia, Colombia); 5 Transdisciplinary Research Group on Socioecological Transitions (GinTRANS2), Universidad Autónoma Madrid, Spain; Ege University, School of Medicine, TURKEY

## Abstract

**Background:**

Increasing evidence shows that health inequalities exist between and within countries, and emphasis has been placed on strengthening the production and use of the global health inequalities research, so as to improve capacities to act. Yet, a comprehensive overview of this evidence base is still needed, to determine what is known about the global and historical scientific production on health inequalities to date, how is it distributed in terms of country income groups and world regions, how has it changed over time, and what international collaboration dynamics exist.

**Methods:**

A comprehensive bibliometric analysis of the global scientific production on health inequalities, from 1966 to 2015, was conducted using Scopus database. The historical and global evolution of the study of health inequalities was considered, and through joinpoint regression analysis and visualisation network maps, the preceding questions were examined.

**Findings:**

159 countries (via authorship affiliation) contributed to this scientific production, three times as many countries than previously found. Scientific output on health inequalities has exponentially grown over the last five decades, with several marked shift points, and a visible country-income group affiliation gradient in the initiation and consistent publication frequency. Higher income countries, especially Anglo-Saxon and European countries, disproportionately dominate first and co-authorship, and are at the core of the global collaborative research networks, with the Global South on the periphery. However, several country anomalies exist that suggest that the causes of these research inequalities, and potential underlying dependencies, run deeper than simply differences in country income and language.

**Conclusions:**

Whilst the global evidence base has expanded, Global North-South research gaps exist, persist and, in some cases, are widening. Greater understanding of the structural determinants of these research inequalities and national research capacities is needed, to further strengthen the evidence base, and support the long term agenda for global health equity.

## Introduction

Globally, there is ever growing interest in health inequalities, and with this there has been an increasing volume of research, which identifies that avoidable systematic differences in the health status of a society exist between and within societies, at all levels (i.e. countries, regions, neighbourhoods) [1–3]. This research has been produced in the context of different global and historical trends in the theoretical and methodological approaches used for the aetiology of health inequalities and their social mechanisms [4,5].

Language and linguistics matters [6], and consequently, the terminology used in this research field has differed over time, as well as between countries and regions. However, most terms share a common element of descriptively identifying a systematic difference in health status between social groups that are unnecessary and avoidable, whilst some go beyond this by emphasising the unfair and unjust nature of these differences [7–9].

Whilst public health research on this topic can be traced back to 19th century [10,11], global interest in health inequalities has consistently grown in the last three decades, and particularly since the establishment of the World Health Organization (WHO)'s Commission on Social Determinants of Health (CSDH) in 2005. The CSDH started to gather global evidence to inform effective action and address 'avoidable' health inequalities [2]. While there was collective agreement at the time, that the CSDH approach—which focused on the Social Determinants of Health (SDH) perspective [12]—provided a necessary alternative to the biomedical and individual determinants of health inequalities paradigms, many scholars in the Global South [13,14] and Global North [9,15,16], have further criticised the conceptual and epistemological reductionist approach taken by the CSDH, and in the subsequent mainstream health inequalities literature, that has predominately concentrated on the North's experience of these inequalities [10,17,18].

Many social scientists have discussed the historical and persistent undervaluing of scientific knowledge generated in the Global South, which is thought to include Eurocentric, Anglo-Saxon and Neo-colonialist tendencies, in the production and evaluation of research, as well as in authorship [5,6,17,19–22]. Thus, in the case of health inequalities research, if a dominant focus is on the Global North's experience and understanding of these inequalities, this may then feed an assumption that the Global North's scientific approaches may be methodologically more developed in their attempts to answer the question of *how to achieve population health equity*? [6];[23] and to define and guide global action [22], and indirectly reduce the Global South to a peripheral player and data-gathering source, rather than an active, research peer [19,20,24,25].

The CSDH 2008 final report '*Closing a gap in a generation'* [2] set an aspirational goal of a narrowing the health gaps that exist at all levels of society, and included three main overarching recommendations for action; one in particular was focused on the need to understand and measure the problem and impact of action [2]; [26], based on the dominant assumption that evidence provides the basis for action [16];[27]. Within this overarching recommendation, included the need for dedicated efforts to strengthen and share the global evidence base on health inequalities, expand the scope of public health research, and to develop dedicated trained workforce and information systems, as well as to raise public awareness—all to improve the capacity to act and address health inequalities.

At the same time, in the global health and development agenda over the past three decades, there has been a strong emphasis on capacity building and strengthening national health research systems, with a need for more country-specific research, particularly from the Global South, which has seen a rise in international research partnerships—all to support a more equitable, global presence in the production and utilization of research for action [2,28–32].

Research capacity, refers to the potential to effectively use resources in order to produce research, and the analysis of health research capacity has become a specific object of study in itself, to determine what kind of capacity exists, where, by whom, and what needs to be strengthened [30,33–35] and this has begun to be examined for health inequalities research, specifically [17,36–42].

Scientific output is considered a crude indication of research capacity, as it is a comparable source that can indicate the amount of research that has been undertaken, where, and by whom. Bibliometric analysis is a useful quantitative tool that can measure and evaluate trends of scientific output, and as such is increasingly used to support evidence informed decision-making processes [43]. Despite its wide application to the health research field, so far this tool has been limitedly applied to the health inequalities research field [44–46].

Nevertheless, these previous analyses show the current lack of global bibliometric knowledge on health inequalities research, and even suggest that systematic differences (potential inequalities) exist in the scientific production on health inequalities. For example, Almeida-Filho et al.[45] found that 75% of the total Latin American and Caribbean (LAC)'s regional scientific production on this topic during 1971 to 2000, was concentrated mainly in four countries, and considered there to be only three country 'epicenters' for this type of research in the region; regional results are discussed collectively in the article, but only a few countries were discussed in detail, and there has been no updated regional analysis since. In addition, Bouchard et al. [46] found 56 countries had contributed to this research field during 1966 to 2014, 10 of which contributed to 94% of this production; however, the results only mention a handful of the top contributing countries, all of which were examples from the Global North.

As such, these results enabled us to pose some important research questions, such as what is known about the global and historical scientific production on health inequalities to date? How is it distributed, in terms of country income groups? What has emerged from different countries and regions, especially those not previously studied? How has this changed over time? What type of research networks and dynamics exist within this global scientific output–to establish which countries are the most influential concerning their contributions to the international journals in this field? And, do inequalities in fact exist in this research field, globally?

The aim of this study is thus fourfold: i) to analyse the volume of global scientific production on health inequalities for over a half of a century (1966–2015); ii) to analyse the distribution of this scientific production by country income groups and world regions, iii) to analyse the international collaborations (e.g. co-authorship relations) within this production, and iv) to establish whether inequalities do exist within global health inequalities research.

## Methods

### Data source

A bibliometric analysis of scientific publications on health inequalities was conducted over a 50-year period (1966–2015). To accommodate the expected lag in the indexing of publications into the bibliometric databases, publications from 2016 were not included in the analysis.

Health inequalities research is known to be transdisciplinary, with health inequalities having been analysed from most scientific fields; for this purpose, Scopus database was selected as the best choice since it allows for bibliometric analysis (similarly to Web of Science, but unlike PubMed), and it offers more journal coverage than Web of Science [47].

### Search strategy

To ensure high sensitivity of the results, our theoretical and methodological approaches considered the historical and global evolution of what is now identified and understood to be research on health inequalities [5];[48], and the following comprehensive search strategy was defined to:

**[Title, Abstract or Keyword]:** (health inequ*) **OR** (health equal*) **OR** (health equity) **OR** (health disparit*) **OR** (health/ disparit*) **OR** (health/ inequ*) **OR** (disparit*/ health) **OR** (ineq*/ health) **OR** (equit*/ health) **OR** (equal* /health) **OR** (inequ*/ mortality") **OR** (disparit*/ mortality) **OR** (social /gradient / health) **OR** (poverty/ health) **AND** (1 January 1966–31 December 2015) **AND Doctype** (Article/ Review/ Editorial).

The 'fixed-term' search terms (health inequ*), *(health equity)*, (health disparit*) and (health / disparit*) were used to retrieve publications referring to (and including the terms) health (and/or status) inequalities for example, and that accounted for the different terminology used in the literature. The semi-free-text search terms (*poverty / health*) and (*social / gradient / health*) were chosen to retrieve historical publications that analyse relationships between poverty and health outcomes, and those that identify and describe the different gradients in health or health inequalities according to social (socioeconomic) stratification; followed by (inequ* or disparit* / health) to retrieve publications that try to understand the potential causes and/or mechanisms (acting through the multiple axes of social position) that generate health inequalities e.g. social inequalities of health or disparities in healthcare access [5];[48]. Additionally, the semi-free-text search terms (inequ* or disparit* / mortality) was chosen to retrieve publications that examine different eco-social or socio-demographic inequalities in mortality outcomes.

In order to capture both the CSDH and Latin-American perspectives, the terms SDH and Social Determination of Health [49] were also considered and other related search terms tested, however through random sampling, we established that the relevant publications could be captured through the use of the other search terms; therefore, no additional search terms were included.

The semi-fixed text search term (health variation*) was also considered to potentially retrieve publications from the United Kingdom (UK) specifically, since under the conservative governments of Margaret Thatcher and John Mayor in the 1980s and early 1990s, the neutral expression “health variations” was deliberately promoted in place of “inequalities” in health [50]. However, after applying this search term and screening all retrieved results from the entire period, the majority of the publications were found to be false positives (i.e. publications retrieved through the search, by the search term(s), but that were not actually relevant to the topic of interest), and the search term was thus excluded from the final search strategy.

Due to the sheer volume of publications retrieved was impractical to hand-search them all, to validate the approach two authors hand-searched all publications from 1966–1990 as well as a random sample from 1990–2015, testing individual search terms and combined search terms, and any uncertainties were discussed between two authors.

### Selection criteria

Inclusion criteria were all publications during the period from 1 January 1966 (when the first bibliometric database was created) to 31 December 2015 (1966–2015); with the search terms mentioned in the Title, and/or Abstract and/or Keywords; document type was restricted to original articles, reviews and editorials; geographical or language restrictions were not applied. Publications from unrecognized or former countries, or with any incomplete author affiliation indexed information, were omitted from the distribution related analysis.

### Data processing

Data on authors country of affiliation and year of publication was exported from Scopus database (March 2017). As country income group can be an indication of the potential size of its national budget for research, author’s country of affiliation were classified by income group (HIC: High income countries, UMIC: Upper middle income countries, LMIC: Lower middle income countries, LIC: Low income countries) according to World Bank classification [51].

Publications were classified into country income group, according to the affiliation reported by each author. Multiple affiliations were considered, so publications can be assigned into more than one income level. For each country income group, analyses were only performed on periods where at least one publication per year was reported.

Furthermore, author’s country of affiliation were also classified by world regions (seven regions), according to World Bank classification [51]. Data on Gross Domestic Product (GDP) per capita, 2015 or latest year, (current USD- Dollars, World Bank database updates as of 1/02/2017), and population size (2015) was obtained from the World Bank as primary source [51] or the World Fact Book as a second choice [52].

We analysed both the related country income group and geographical distribution of scientific production, which quantifies the volume of scientific production on health inequalities that each country has contributed to, according to the authors' country of affiliation at the time of publication (i.e. country is the unit of analysis).

It should be noted that this does not necessarily represent the original nationality of the author, however it is the best proxy indication available for country contribution, and if a foreign author signs their affiliated to a certain institution in a certain country, then the implied assumption for the analyses was that they may be considered as a “member” of the scientific community of that country.

Publications where co–authorship was international, were counted more than once, therefore the sum of the number of publications per country (income group and world region) does not directly correspond to the overall volume of production retrieved, but reflects the participation and contribution of each country to global health inequalities research.

National scientific production refers to the ability of a country to perform certain research outputs, which measured alone, may indirectly represent a number of potential factors, such as level of investment in research, population size or the presence of institutional support. Whereas national scientific productivity, refers to the ability to achieve research outputs, whilst also considering the available resources (e.g. research co-authored per human, financial or technical unit), of lack thereof; thus we also calculated proxies of national scientific productivity by country population size and GDP per capita [53]. For example, calculating Brazil's health inequalities scientific productivity per GDP per capita = volume of historical health inequalities scientific production (n = 737)/ Income of country (i.e. 8757.21 USD GDP per capita, 2015) = 0.08 co-authored publications by GDP per capita. For example, calculating Brazil's health inequalities scientific productivity by total population = volume of historical health inequalities scientific production (n = 737)/ total population (n = 205.96 million) = 3.6 co-authored articles per million population.

### Data analysis

#### Volume of scientific production

Our study analysed the annual volume of global scientific production on health inequalities (1966–2015); joinpoint regression analysis was undertaken to examine the time trends in health inequalities scientific production over the last half a century, by country income group.

The number of publications was set as the dependent variable; the year was set as the independent variable. Constant variance for error terms was assumed. We considered independent models with 0 to 3 joinpoints, and used permutation tests to identify the best fitting number of statistical different periods to describe time trends for each income level group. Additionally, Average Percent Change and its statistical significance was estimated to summarize and compare the magnitude of intra-period changes by country income groups; a p-value ≤0.05 was considered statistically significant. As initiation and consistency in publication frequency differed by income group, joinpoint analyses were performed starting in the year from which at least one publication per year was reported. Analysis were performed in JoinPoint Regression Program [54].

#### Distribution of scientific production

We analysed both the income related and geographical distribution of scientific production, which quantifies the volume of scientific production on health inequalities that each country has contributed to.

The percentages of publications that include at least one author from the different country income groups and each world region were also calculated, to show the publication distribution between different country income levels and world regions. We presented only the first 20 country contributors per income group.

Additionally, for each of these categories the percentage of publications, with first authors' country affiliation only to countries within the same income group, and the same world region, were calculated and compared; this process was independently performed for first author and for all authors within each publication. All-authors estimates include the first author. Furthermore, we calculated proxies of national scientific productivity, by country population size and GDP per capita.

#### International collaborations and co-authors network relations

Our study analysed the strength of international collaborations within health inequalities research, through the analysis of co-author networks [55]. We used VosViewer software 1.6.5 [56] to create two types of bibliometric network visualization maps, which depict the publications co-authored by each country of affiliation relating to i) the cluster's link strength network within this global research activity (Map) and ii) the individual countries link strength network (i.e. inter-country co-author relation) within these clusters (Map 2).

The cluster's link strength network map (Map 1), highlights the separation between main clusters by density measures. In the individual countries link strength network map (Map 2), location and colour are identical, but proximity must be evaluated by visual inspection. As distance metric is not intuitive, the inclusion of density measures tries to support this process.

Within both network maps, the different colours represent different clusters memberships within these two network levels, based on link strength. Centrality in the maps is relevant, as it represents core countries. Relative proximity is also relevant, for example, the smaller the distance between the i) clusters or ii) individual countries, the stronger relation.

In addition, for the countries link strength network map (Map 2), each country affiliation is represented by a circle, the size of a circles indicates the total links (co-author activity) of the country, and the lines between countries represent bi-national co-authorship links, and the thickness of the line represent the strength of the co-authorship inter-country relation.

## Results

We initially retrieved 33,954 scientific publications on health inequalities (1966–2015), of these, 4,575 publications were then excluded as co-authors country affiliations were undefined. A final total of 29,379 scientific publications were then used in the data analysis, the majority of which were original articles, followed by reviews, and editorials.

### Volume of scientific production

**Fig 1:**
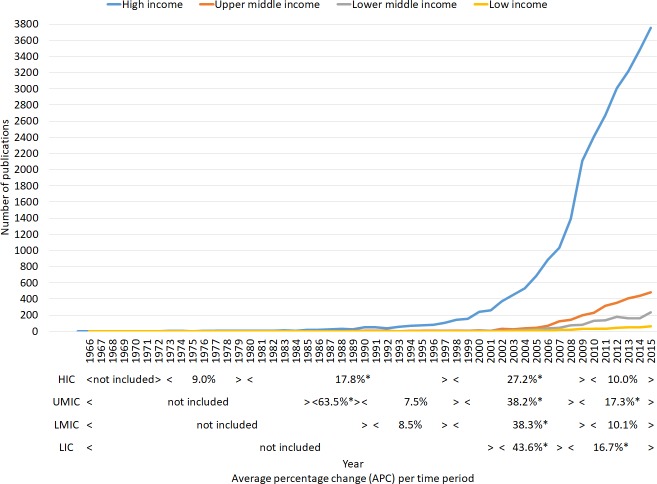
Global health inequalities research time trends, by income group of authors’ country of affiliation (1966–2015). Footnote: (><) = as approximate positions of the joint points; * = p<0.05.

According to our results, the volume of scientific production on health inequalities has exponentially grown over the last five decades (**Fig 1**).

The first publication dates back to 1966, however it was not until the early 1970's (1973–1979) that publications begin to appear annually (e.g. with at least one publication per year reported), visible by the appearance of the first joinpoint period of analysis, but specifically by HIC affiliations only. It was not until the early 2000's, that publications began to appear annually by LIC affiliations. We found a visible country-income group affiliation gradient in the initiation and consistent publication frequency on health inequalities.

There are also important similarities in the position of the last two jointpoints (e.g. around the same years) for all income groups, Between 1997 and 2002, and between 2007 and 2010, statistical significant changes in time trend were observed for all income groups, with a consistent increase in publication frequency (**Fig 1**).

For HIC author affiliations specifically for example, consistent co-authorship in health inequalities research started in 1973, and showed four periods (three joinpoints) with different time trends: period one (1973–1979) with non-significant average change in co-authored publications, periods two (1980–1997) and period three (1998–2009) when co-authored publications annual average growth were 17.8% and 27.2%, and, period four (2010–2015) with non-significant Average Percent Change.

### Distribution of scientific production

We found that 159 countries contributed to the global scientific production on health inequalities during this 50-year period. The top 20 countries that have contributed the most to global health inequalities research field were established, as well as each country proportional contribution to the total research output, and scientific productivity, considering both GDP per capita, and population size. Of these 20 countries, 16 were HIC from North America, Europe and Central Asia, and East Asia and the Pacific Regions, two UMIC from LAC, Sub-Saharan Africa, and East Asia and the Pacific Regions, and one LMIC from South Asia. We also established the top 20 countries contributors per country income group, as well as their proportional contribution, and scientific productivity by population size and GDP per capita (**Tables 1–4**).

**Table 1:** Top 20 high income country contributors to global health inequalities research (1966–2015), ranked by co-authorship affiliation.

HIC rank	Global rank	Country of co-authorship affiliation	Volume of health inequalities scientific production (n)	Proportional Co-authorship contribution to global health inequalities scientific production (%)	Health inequalities scientific productivity per GDP per capita	Health inequalities scientific productivity per million population
1	1	United States	16495	48.58	0.29	51.4
2	2	United Kingdom	4257	12.54	0.10	65.4
3	3	Canada	2116	6.23	0.05	59.0
4	4	Australia	1650	4.86	0.03	69.4
5	5	Netherlands	741	2.18	0.02	43.8
6	7	Germany	713	2.10	0.02	8.7
7	8	Sweden	673	1.98	0.01	68.7
8	9	France	663	1.95	0.02	10.0
9	10	Spain	623	1.83	0.02	13.4
10	11	New Zealand	518	1.53	0.01	112.7
11	12	Switzerland	453	1.33	0.01	54.7
12	13	Italy	418	1.23	0.01	6.9
13	15	Norway	381	1.12	0.01	73.5
14	18	Belgium	334	0.98	0.01	29.6
15	19	Finland	311	0.92	0.01	56.8
16	20	Denmark	292	0.86	0.01	51.4
17	21	Japan	253	0.75	0.01	2.0
18	22	South Korea	244	0.72	0.01	4.8
19	24	Israel	183	0.54	0.01	21.8
20	25	Ireland	166	0.49	0.0	35.5

**Table 2:** Top 20 upper-middle income country contributors to global health inequalities research (1966–2015), ranked by co-authorship affiliation.

UMIC rank	Global rank	Country of co-authorship affiliation	Volume of health inequalities scientific production (n)	Proportional Co-authorship contribution to global health inequalities scientific production (%)	Health inequalities scientific productivity per GDP per capita	Health inequalities scientific productivity per million population
1	6	Brazil	737	2.17	0.08	3.6
2	16	South Africa	362	1.07	0.06	6.6
3	17	China (ex. H Kong)	347	1.02	0.04	0.3
4	23	Mexico	221	0.65	0.02	1.8
5	28	Iran	129	0.38	0.03	1.6
6	31	Thailand	104	0.31	0.00	1.5
7	35	Argentina	91	0.27	0.01	2.1
8	45	Turkey	68	0.20	0.01	0.9
9	49	Peru	57	0.17	0.01	1.8
10	50	Malaysia	54	0.16	0.01	1.8
11	54	Slovenia	44	0.13	0.00	21.3
12	57	Estonia	40	0.12	0.00	30.4
13	60	Romania	37	0.11	0.00	2.0
14	62	Lebanon	35	0.10	0.00	6.0
15	68	Cuba	27	0.08	0.00	2.4
16	70	Serbia	23	0.07	0.01	3.2
17	71	United Arab Emirates	22	0.05	0.00	2.4
18	78	Bulgaria	18	0.05	0.00	2.5
18	78	Costa Rica	18	0.05	0.00	3.7
20	81	Georgia	15	0.04	0.00	4.1

**Table 3:** Top 20 lower-middle income country contributors to global health inequalities research (1966–2015), ranked by co-authorship affiliation.

LMIC rank	Global rank	Country of co-authorship affiliation	Volume of health inequalities scientific production (n)	Proportional Co-authorship contribution to global health inequalities scientific production (%)	Health inequalities scientific productivity per GDP per capita	Health inequalities scientific productivity per million population
1	14	India	404	1.19	0.25	0.3
2	27	Colombia	139	0.41	0.02	2.9
3	30	Kenya	111	0.33	0.08	2.3
4	38	Nigeria	85	0.25	0.03	0.5
5	44	Pakistan	69	0.20	0.05	0.4
6	47	Ghana	65	0.19	0.05	2.4
7	48	Bangladesh	64	0.19	0.05	0.4
8	51	Vietnam	50	0.15	0.02	0.5
9	56	Egypt	40	0.12	0.01	0.4
10	58	Philippines	39	0.11	0.01	0.4
11	64	Indonesia	34	0.10	0.01	0.1
12	72	Zambia	22	0.06	0.02	1.4
13	74	Congo (Dem. Rep)	21	0.06	0.04	0.3
14	76	Sri Lanka	19	0.06	0.00	0.9
15	81	Guatemala	15	0.04	0.00	0.9
16	84	Tunisia	14	0.05	0.00	1.5
16	84	Morocco	14	0.04	0.00	0.4
18	88	Sudan	13	0.04	0.01	0.3
19	92	Nicaragua	12	0.04	0.01	2.0
20	96	Senegal	11	0.03	0.01	0.7

**Table 4:** Top 20 low income country contributors to global health inequalities research (1966–2015), ranked by co-authorship affiliation.

LIC rank	Global rank	Country of co-authorship affiliation	Volume of health inequalities scientific production (n)	Proportional Co-authorship contribution to global health inequalities scientific production (%)	Health inequalities scientific productivity per GDP per capita	Health inequalities scientific productivity per million population
1	43	Tanzania	70	0.21	0.08	1.3
2	46	Uganda	68	0.20	0.10	1.7
3	61	Malawi	36	0.11	0.10	2.0
4	65	Nepal	33	0.10	0.04	1.2
5	66	Ethiopia	29	0.09	0.04	0.3
6	73	Burkina Faso	21	0.06	0.04	1.2
7	75	Cambodia	20	0.06	0.02	1.3
8	80	Laos	16	0.05	0.01	2.4
9	81	Rwanda	15	0.04	0.02	1.3
10	84	Mozambique	14	0.04	0.03	0.5
11	88	Zimbabwe	13	0.04	0.01	0.8
12	101	Gambia	8	0.02	0.02	4.0
13	107	Haiti	6	0.02	0.01	0.6
14	112	Sierra Leone	5	0.01	0.01	0.7
15	124	Afghanistan	4	0.01	0.01	0.1
15	124	Mali	4	0.01	0.01	0.2
17	133	Benin	2	0.01	0.0	0.2
17	133	Guam	2	0.01	-	12.3
17	133	Guinea	2	0.01	0.0	0.2
17	133	Guinea-Bissau	2	0.01	0.0	1.1

The top HIC contributor in terms of scientific output was the United States, which alone contributed to 48.5% of the global scientific production on health inequalities, with at least one author affiliation in each publication, and the Anglo-Saxon countries, with the United States, UK, Canada and Australia combined having contributed to ~ 70% of this scientific production, with at least one author affiliation from these countries (**Table 1**).

Brazil was the top UMIC contributor, having contributed to 2.2% of the global scientific production, India was the top LMIC contributor having contributed 1.2%, and Tanzania was the highest LIC contributor, having contributed 0.2% (**Tables 1–4**).

**Fig 2:**
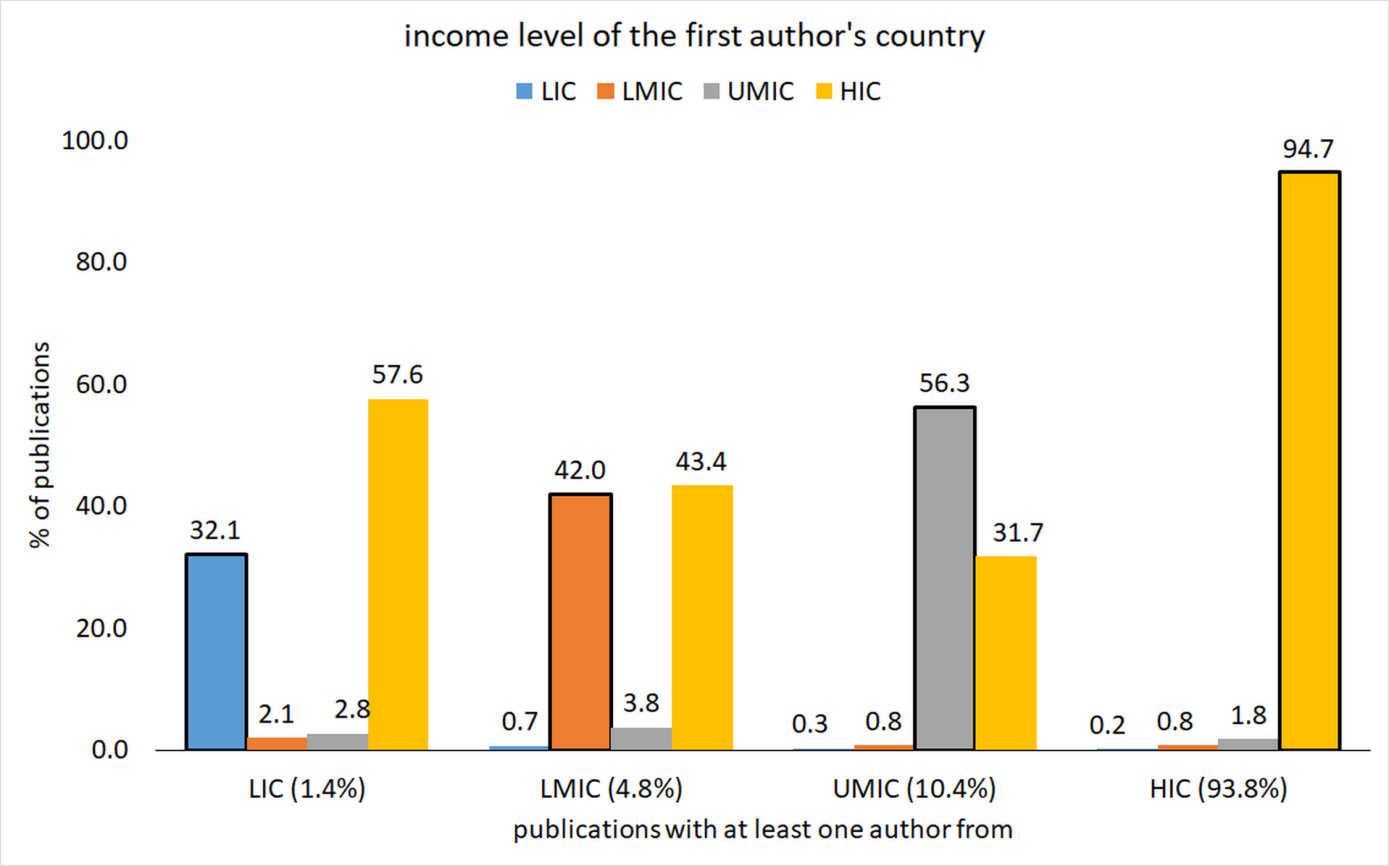
Global health inequalities research by income group of first authors' and Co-Authors' country of affiliation (1966–2015).

With respect to the proportional distribution of authors and first author country of affiliation, the higher the country income group of author’s affiliation, the higher the proportional distribution of authors (visible by the percentages of publications in the horizontal axis bar) (**Fig 2**).

We observe that amongst the publications with co-authors affiliated to HIC and UMIC, the higher the proportional distribution of first author’s affiliated to that same income group. However amongst the publications with LMIC and LIC affiliations, the distribution of first authors was disproportionally higher for HIC affiliations than from LMIC, and even more so compared to LIC visible by the bold-line bars.

The proportional distribution of LIC first authorship appears to decrease with increasing income group of affiliation of co-authors (visible by the blue bars in each income group) (**Fig 2**).

**Fig 3:**
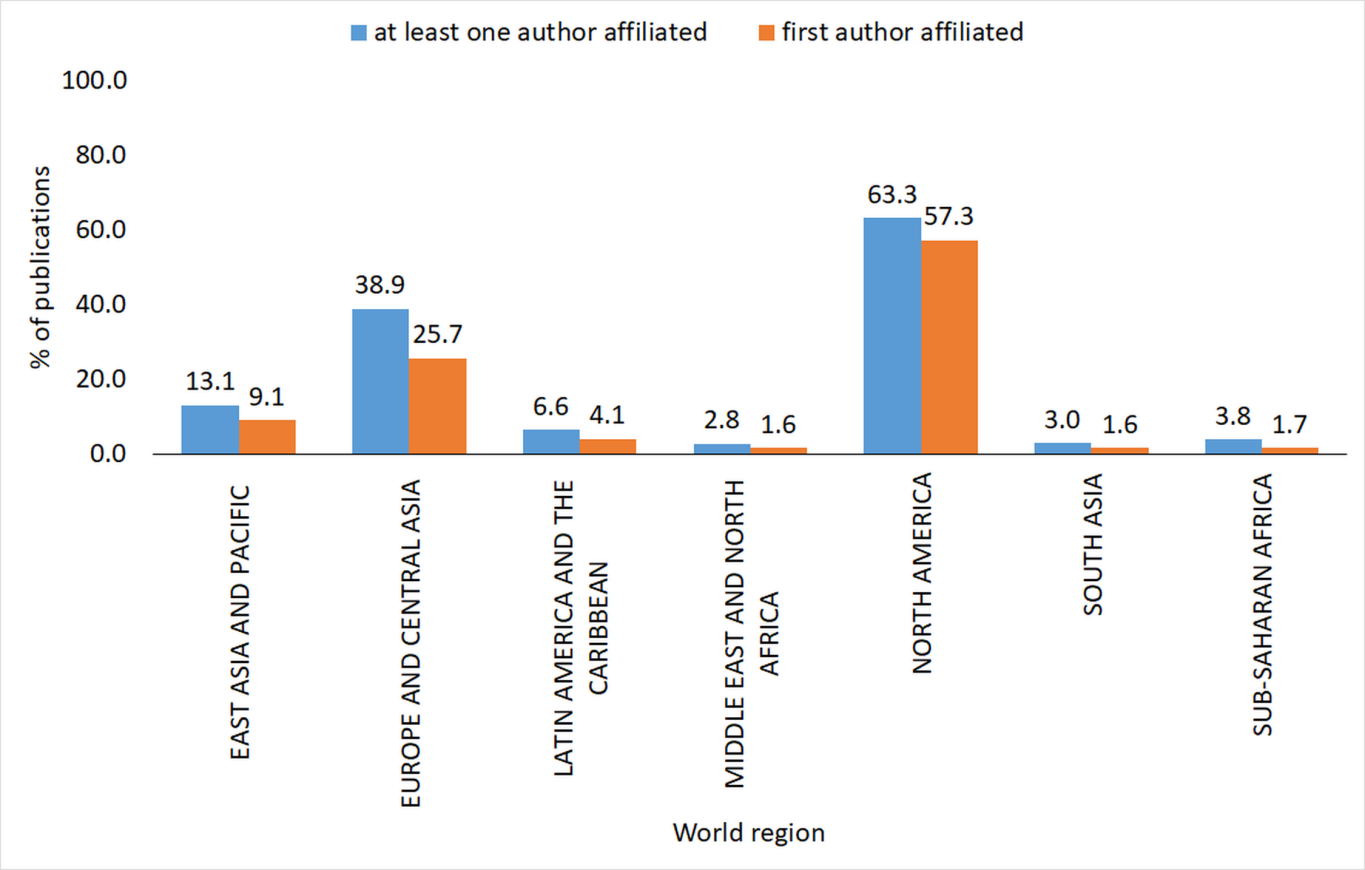
Global health inequalities research by world region of first authors' and Co-Authors' country of affiliation (1966–2015).

With regards to proportional distribution by world region, the higher proportional distribution of co-authors region of affiliation, the higher the proportional distribution of first authors region of affiliation, and world regions which included Anglo-Saxon countries (e.g. North America, Europe and Central Asia, and East Asia and Pacific regions), had the highest proportional distribution of both co-authors and first authors' country of affiliation, compared to other regions (**Fig 3**).

North America had the highest proportional distribution of both co-authors and first authors' country of affiliation, followed by Europe and Central Asia, East Asia and the Pacific, then LAC, Sub-Saharan Africa, South Asia and then the Middle East and North Africa (**Fig 3**).

### International collaborations and co-author networks

**Fig 4:**
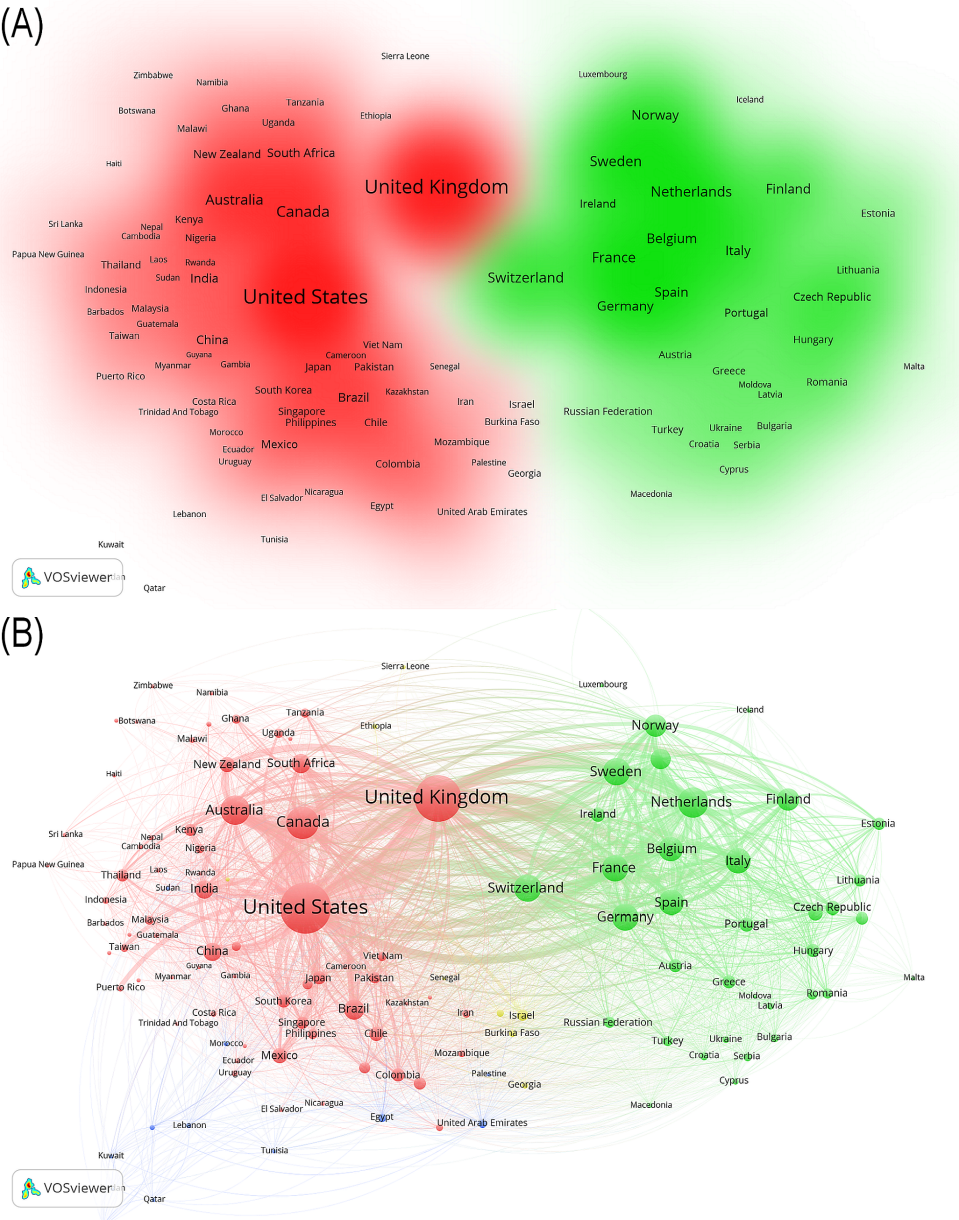
(a-b). Global co-authorship network of health inequalities research (1966–2015). (A) Density visualization of cluster’s link strength. (B) Network visualization of countries’ link strength.

The main network clusters (**Fig 4A**) and individual inter-country relations within and between these network clusters (**Fig 4B**) were also depicted within the global health inequalities research field (1966–2015).

Four clusters were identified based on countries’ total link strength; kernel density colour gradient shows a marked difference between country clusters (**Fig 4A**). Two small clusters with minor participation in global production in health inequalities research formed mainly by Middle East and North African Countries (blue) and by Sub-Saharan Africa countries (yellow), and two main clusters, one led by UK, the United States, Canada and Australia and formed mainly by non-European countries (red), and the other one led by Netherlands and Sweden and formed mainly by European countries (green). The rest of the countries from different world regions (e.g. LAC, South Asia, Sub-Saharan Africa), appears to orbit around these core countries (with different regions) with stronger links with the United States, followed by the UK, than with Europe and Central Asia (**Fig 4A and 4B**).

Bi-national links (**Fig 4B**) show that the UK, at the core of international collaborations, plays a central role at bridging these two main clusters (red and green); however it is classified as a member of the red cluster because the UK-United States shows the strongest link (503 points) followed by US and Canada (493 points). UK is also strongly linked to Canada (225 points) and Australia (259 points).

In relation to European countries (green cluster), the UK strongest links were observed with Netherlands (175 points) and Sweden (148 points). However, their link strength is less than half of the UK-North America link. Besides the UK related links, strong links were also observed between United States and Switzerland (141 points), Germany (131 points) and Netherlands (115 points).

Other relevant non-North America-Europe inter world-region links were identified for Australia (-United States 223 points, -UK 259 points, -Canada 149 points), Brazil (-United States 139 points,—UK 67 points), Mexico (-United States 114 points), China (-United States 140 points), India (-United States 99 points, -UK 88 points), and South Africa (-United States 102 points, -UK 91 points).

Moreover, by cross checking study results (**Fig 4B** with **Tables 1–4**), it is potentially possible to establish whether a country has more domestics vs. international collaborations within the publications that they have contributed to, relative to other countries.

## Discussion

Our study comprehensively analysed the historical and global scientific production on health inequalities research (1966–2015), and clearly demonstrates the magnitude of asymmetric trends, inequitable systematic differences, and potential global dependencies that exist and persist within this research field.

Whilst there has been an exponential increase in health inequalities scientific output globally during this 50-year period, Global North-South research gaps still exist, and may even be widening with respect to country income group. HIC disproportionally dominate co-authorship and first authorship contributions, with the Global North positioned at the core of the global collaborative research networks, with the rest of the world (i.e. Global South) on the periphery of this activity.

### Volume of production

Over the past five decades, the volume of health inequalities scientific output has grown exponentially, more so than the average trends in scientific output in general [57], likely linked to the increased interest and sophistication in the analysis and understanding of health inequalities over time [5];[48], and how they are the social consequence of a hegemonic eco-political agenda that only benefits narrow class interests [9]. However, within these trends there is a visible country income level affiliation gradient with respect to the initiation in the production of the first publication and in the consistent frequency in production, where authors' affiliated to HIC started to publish first (during the 1960s), and more frequently (during the 1970s), as well as in the designation of first authorship.

We found several notable shift points in the volume of research production, firstly around the late 1980's-90s there was a significant, consistent increase in publications for HIC and UMICs, coinciding with the government interest and awareness of how social conditions and material deprivation shape health inequities and mortality, firstly in the UK [58–60] the United States [61,62] and Canada [63], later followed by European counties [64], which most likely drove further research interest on this topic in these countries and regions. Alongside this, has been the long research tradition in Social Medicine and Collective Health within Latin America, studying the link between social (and power) inequalities and health, that traditionally has not been as widely known or acknowledged outside of the region [6], and which appeared to enter the international scientific literature around this time.

There was a significant, consistent increase in publications for all income groups in the early 2000s, a likely consequence of the rise in social justice and 'equity in health' disource in the 1990s, that acknowledged the need to assess both economic development of countries and human welfare [65]. This, in combination with the increased sophistication in the analysis of these issues, created new perspectives on health and well-being, which likely fed into the shifts in the global health and development agendas, which we described in the introduction, and that subsequently led to the establishment of CSDH in the early 2000s.

A few years later, another significant and consistent increase in publications also occurred, again for all income groups, likely coinciding with CSDH's final report (2008) and its recommendations that emphasised the need for further global research on this topic, and the additional importance placed on addressing health inequalites [2]. Since that point, there was another significant consistent increase in publications, for authors affiliated to UMIC and LIC income groups specifically.

By 2015, there appeared to be a difference of around 25 years between the volume of research production by authors affiliated to LIC, compared with when authors affiliated to HIC reached this same volume of production (i.e. during the 1990s). The visible country income related gradient and time difference in volume of production, may partly be explained by the fact that traditionally, there has been a high publishing and subscription costs for international journals, which may have impeded some lower income countries research publication process, even despite the later open access movement and reduced costs for lower income countries [16]; [37], amongst other things.

Another possible explanation, linked to the CSDH 2008 report recommendations, is that in general lower income countries have more limitations in their national health information and surveillance systems, which reduces the capacity to collect, monitor and analyse reliable health and social-demographic data, and subsequently hinders the capacity to produce research on the social determinants of health inequalities within a national context.

### Distribution of scientific production

We found that 159 countries have contributed to this global scientific production indexed in Scopus, which equates to 86% of the world. When examining the proportional contributions of country (corresponding to author affiliations) to the global research base, the following Anglo-Saxon countries—The United States, UK, Canada and Australia—combined have contributed to around 70% of this scientific production, with at least one author affiliation from these countries. The European and Central Asian region collectively, has contributed to approximately 33% of this scientific production.

Bouchard et al. [46], previously found notably more health inequalities publications during 1966–2014 (n = 49,294) than our study, yet only found 56 countries had contributed to the global scientific production. These important differences are likely due to the differences between the theoretical and methodological approaches used in our studies. For example, Bouchard et al. [46] do not state the theoretical assumptions used to inform their approach, although, based on the search strategy, they appear to have a slight tendency towards "*health care (e*.*g*. *Medicare)"* inequalities (p101), which would have likely led to the retrieval of false positives into the initial search results; whilst healthcare services are linked to health inequalities, the health-care system itself is considered just one of many intermediary determinants which can be influenced by, and influence the effect of, other determinants of health inequalities [4].

However, our theoretical and methodological approach specifically considered the historical and global evolution of, what is now identified and understood to be, research on health inequalities, produced by different countries and over time, to ensure a high specificity in our retrieve process. This likely explains how, even though we retrieved fewer publications (potentially due to our search strategy document type restrictions), even with the inclusion of one extra year in our analysis, we retrieved publications from 103 more country affiliations than Bouchard et al. [46].

### Scientific productivity

Distinguishing between the proportional contributions to the global health inequalities research, by income group and world regions, not only helps to better understand the global research landscape, but it can potentially allow for fairer country comparisons to be made amongst countries with similar resources levels and geo-cultural perspectives, and moves one step closer to a deeper understanding of the potential reasons behind the different national levels of scientific production on this topic. This type of disaggregated information may also be useful to consider when conceiving future Global South-South and Global North-South research collaborations and partnerships within this research field.

Furthermore, when national health inequalities research output was adjusted by socio-economic and socio-demographic country characteristics (i.e. scientific productivity), we found several countries actually perform particularly well, despite their limited resources; these results are similar to what Bahenhorst et al. [66] found for public health research more broadly. For example, when scientific production on health inequalities was adjusted by country income (GDP per capita), Uganda and Malawi perform equally well as the UK, and when adjusted by population size, Estonia performs better than Germany.

We would have liked to have adjusted national scientific output by the proportion of GDP expenditure on Research and Development (R&D), however challenges exist regarding governance and capacity to collect and report this type of data consistently, across years [67], and for all countries [68]. However, the WHO Global Observatory on Health R&D uses the limited data available to show general trends, and so we considered these findings with respect to our study results [69]. These general trends show that, on average, HIC have 3524 health researchers full-time-equivalent per million inhabitants compared to UMIC which have 885, LMIC which have 53, and LIC which have 10 full-time-equivalent per million inhabitants on average [69].

These trends, as well as our study results, do not of course account for '*brain drain*', the migration of trained professions, mainly from the Global South to Global North, which translates into a considerable loss of resources that were invested into the trained professionals by the home country, that the recipient country then benefits from [70]. Nevertheless, these results do provide an indication of potential human resource availability within countries; the presence of a trained national work force can strongly influence national scientific output and is another important component and/or determinant of national research capacity [33]. These results may partly explain the income related differences in national research output that we found.

At regional and country level, there are some interesting cases, specifically from the Global South, worth highlighting. For example, Brazil was the 5^th^ global contributor of health inequalities research, after the United States, UK, Canada and Australia, and the top LAC regional contributor, followed by Mexico. Brazil is classified as a UMIC, and is well-known for its long research tradition in public health and Social Medicine and Collective Health [71], for its strong political commitment that has contributed to the national mobilization for social and health equity [45];[72], and for its national repository and observatory of health and its social determinants [73,74], which contributes to the on-going systematization of evidence and aims to guide future national research and policy agendas on this topic. All of which, without a doubt, has fostered the countries strong health inequalities research capacity that can be observed here.

Almeida-Filho et al. [45] considered there to be three main regional 'epicenters' for health inequalities research during 1977 to 2000, based in Brazil, Mexico and Chile, with Argentina's and Colombia's scientific output being more *"scattered and unstable"*. In 2001, Waitzkin et al. [23] stated that the most favourable institutional conditions for social medicine research in Latin American at the time, existed in Mexico, Ecuador, Brazil and Cuba, and that in Argentina, Chile and Colombia, as the socio-political conditions remained more adverse, researchers faced challenges in producing research in this specific field.

Fifteen years on, we could still identify the regional “epicenters” that Almeida-Filho et al. [45] describe, as well as the strong intra-regional co-author links, potentially due to geographical and socio-cultural proximity, and linguistic relationships, and the strong national and regional interest in this research topic. However, Colombia's volume of health inequalities scientific output increased specifically in the last decade, and by 2015 overtaking that of Chile's. Also, interestingly, the LAC region, especially its 'epicenters', have stronger co-author relations and links with the United States, followed by the UK, than with Europe, the reasons for which, may again firstly be due to geographical proximity (to the United States), and or historical and global geopolitical relationships, which we will elaborate on further in the next section.

Another interesting case is India, positioned as 14th global contributor of health inequalities research, the top LMIC contributor, and the top South Asian regional contributor in this research field. A recent systematic review focusing on health inequalities research production in India over the last 30 years, found that 75% of papers retrieved were led by Indian institutions, and stated that national social and political movements have played an important role in highlighting inequalities, in addition to social medicine developments in public health education, and increased availability in population survey data, which collectively, similarly to the case of Brazil, have likely assist to built strong national capacity for research on health inequalities [75].

### International collaborations and co-author network relations

With respect to the international collaborative research networks, there appears to be a clear distinction between those countries at the core of the global health inequalities research collaborations (e.g. United States and the UK, followed by the other Anglo-Saxon countries, the Nordic and Central-Northern European countries), and those on the periphery of this activity (e.g. the Global South). Furthermore, the proportional distribution of both co-authors and first authors' country of affiliation were higher for authors affiliated to worlds regions that specifically include Anglo-Saxon countries.

There was also a visible country income affiliation gradient with respect to the proportional distribution of authors and first authors' country of affiliation, with HIC affiliations disproportionally dominating co-authorship in general, and first authorship positions specifically, especially amongst the publications with authors affiliated to lower country income groups.

This underrepresentation of LIC affiliated co-authorship, and first authorship amongst those papers which do include LIC affiliated co-authors, may partly be a result of, what has been described as, 'neo-colonial science' [20]. However, as mentioned previously, if national research infrastructure and human resources research capacities are limited, then the national capacity to produce research will in turn be limited.

It is also important to note that our study only focused on co-authors and first authors, we did not analyse corresponding author affiliation, which provides another indication of research leadership, although we suspect that similar trends and dynamics are likely to exist for corresponding authorship affiliation.

These respective asymmetries in the global scientific output and collaborations appear to mirror the geopolitical hierarchies and the subsequent dependencies and conditionalities that are known to have been created over time; it is surely no coincidence that the countries known for being international funding sources and also the countries at the core of these global research collaborations [76–78] and those known to be more 'dependent' on external research funding are on the periphery. In many lower income countries, the majority of research is externally funded, which may play a role in fostering and influencing the types of domestic vs. international research collaborations that are built, as well as potentially creating donor-driven research agendas that may influence policy agendas, and decisions on national research priorities, which may not necessarily correspond to local population needs [29,30,38,79].

### Study limitations and other possible explanations

Our study is constrained by a number of limitations; firstly, in terms of the study design, we only focused on articles, reviews and editorials that have been published in academic journals indexed in Scopus. Therefore, this study does not presume to fully reflect all of the work produced on this topic, which may have been published in other forms (e.g. books, reports, and national journals). Nor do we claim to present exact numbers in terms of country contributions to the global scientific production, as we have not hand-searched all retrieved publications to confirm their relevance, although we suspect that our results reflect the general trends that exist within the global health inequalities research landscape.

In addition, the primary source of this bibliometric analysis was international academic journals indexed in Scopus, and international journals are known to contain an English language bias, which may skew our results in favours of Anglo-Saxon countries and/or countries were the national research system incentives publishing predominately in these types of journals [36];[45]; some non-Anglo-Saxon countries have national research systems that incentive and prioritise national publishing of research findings, in the native language and in different forms, to facilitate national dialogue and local strategic decision-making [17];[72]. This may reduce the international visibility of the research, and mask the actual volume of research being conducted in these countries, regardless of bibliometric databases increasing their breadth of journal coverage.

There has also been some speculation by scholars, as to whether 'editorial racism' exists in the evaluation and selection of manuscripts for publications in international journals with prejudice against authors from the Global South [21], and Harris et al. [80] show (and measure) the bias by health professionals and researchers, against research produced by LIC in comparison to HIC. Nevertheless, such peer prejudice could be potentially offset by increased investment in research in the Global South, that includes an additional emphasis on solid methodology, research infrastructure, and high quality presentation, in terms of both writing and (English) language skills [21].

Thus, whilst our results are based only on publications in international academic journals, these findings are important to consider, given the weight placed in academia on publishing in international academic journals, and how it is often used to inform decisions regarding international development, policy and research agendas. Furthermore, our results likely allude to the global dynamic within this research field itself.

Lastly, quantitative bibliometric results say nothing about the type of health inequalities research that has been conducted in countries, globally; further research is needed to contextualise our results and provide in-depth insights into the type of theoretical and methodological approaches being used and where, and the national research priorities, as well as enrich current understanding of the historical and structural determinants of theses global bibliometric trends and inequitable gaps in health inequalities scientific output, and collaborative co-author network dynamics.

## Conclusions

Bibliometric analysis is an extremely useful tool despite its focus on international peer review journals, therefore together with our theoretical and methodological approaches taken to identify relevant global publications, and the data analysis used, we have a strong base on which to state that our study presents a comprehensive systematisation of global health inequalities research (1966–2015), as well as the magnitude of the inequitable bibliometric trends and asymmetries that exist, and persist, in this research field, globally.

Whilst there has been an exponential increase in health inequalities research output during this 50-year period, and three times as many countries have contributed to this global evidence base than previously found, Global North-South research gaps still exist, and in some cases are ever widening. Higher income countries, especially Anglo-Saxon and European countries, disproportionately dominate first and co-authorship, and are at the core of the global collaborative research networks, with the rest of the world (i.e. the Global South) on the periphery of this activity. However, several interesting country anomalies exist, that suggest that the causes of these inequalities and potential underlying dependencies within this research field, run deeper than simply differences in country income and language.

Greater understanding of the structural determinants of these research inequalities and national research capacities is needed, so as to strengthen the evidence base on health inequalities, making it more inclusive and globally representative, which can foster more shared learning, and provide more effective support towards the long term agenda for global health equity.

## Supporting information

S1 Dataset Minimal manuscript dataset. (XLSX)
